# Securing MQTT by Blockchain-Based OTP Authentication

**DOI:** 10.3390/s20072002

**Published:** 2020-04-03

**Authors:** Francesco Buccafurri, Vincenzo De Angelis, Roberto Nardone

**Affiliations:** Department of Information Engineering, Infrastructure and Sustainable Energy (DIIES), Mediterranean University of Reggio Calabria, 89124 Reggio Calabria, Italy; vincenzo.deangelis@unirc.it (V.D.A.); roberto.nardone@unirc.it (R.N.)

**Keywords:** Ethereum, Internet of Things, authentication, smart contracts, MQTT, one-time password

## Abstract

The Internet of Things is constantly capturing interest from modern applications, changing our everyday life and empowering industrial applications. Interaction and the collaboration among smart devices offer new challenges to security since they conflict with economic and energy consumption requirement constraints. On the other hand, the lack of security measures could negatively impact the concrete adoption of this paradigm. This paper focuses on the Message Queuing Telemetry Transport (MQTT) protocol, widely adopted in the Internet of Things. This protocol does not implement natively secure authentication mechanisms, which are demanded to developers. Hence, this paper proposes a novel OTP (one-time password)-authentication schema for MQTT, which uses the Ethereum blockchain to implement a second-factor out-of-band channel. The proposal enables the authentication of both local and remote devices preserving user privacy and guaranteeing trust and accountability via Ethereum smart contracts.

## 1. Introduction

The Internet of Things (IoT) is a well-known paradigm that relies on the interactions of intelligent computing devices. Each device (and each user) in the IoT domain is provided with a unique identifier. According to the Reference Architecture reported in the standard ISO/IEC 30141 [1] the devices can be classified as sensors or actuators, and they are connected to the Internet in order to be reached by a generic user of the system. This paradigm had rapid growth in recent years. In fact, according to a Gartner study, we may expect to reach about 50 billion devices by the end of 2020 [2], also thanks to the migration to the IPv6 [3] version of the Internet Protocol, which would solve the problem of IPv4 address exhaustion.

The design and the development of an IoT application must cope both with the problem of limited energy consumption and security issues, among other challenges. These requirements often conflict, and developers must find a compromise when designing novel solutions. Moreover, existing protocols commonly adopted in IoT were designed without facing security issues that are demanded of developers and depend on the specific application. As an example, the Message Queuing Telemetry Transport (MQTT) [4] protocol, one of the widely adopted protocols for communication among devices, is an example of a source of security vulnerabilities. As will be clear in the rest of this paper, MQTT requires the usage of a message broker (i.e., the MQTT broker) to enable the adoption of the publish–subscribe pattern. The broker interconnects a set of clients, playing the roles of publishers and/or subscribers. In the IoT domain and according to the ISO Reference Architecture, the publishers are commonly sensing devices, while subscribers are actuators.

MQTT does not include strong security features. In the MQTT specification, security is completely demanded to developers, increasing the risk of bad implementation. The protocol specification, in fact, highly recommends the adoption of transport layer security (TLS) at the transport layer to enforce security. Focusing specifically on the authentication, the MQTT built-in mechanism is very weak. It is based on the transmission of username and password in a CONNECT message. The latest version of MQTT, i.e., Version 5.0 released in March 2019 [5], still specifies that a username and password are sent in the CONNECT message, respectively as UTF-8 encoded string and binary data. Even if MQTT version 5.0 provides an enhanced authentication mechanism that allows, for example, the usage of the password as a token, it is explicitly stated that the protocol suffers from man-in-the-middle and replay attacks, also in the authentication phase. The confidence of having only authorized clients in the network can be obtained by adopting a virtual private network. Similarly, TLS certificates sent from clients can be used to authenticate them to servers.

This paper focuses on the authentication in MQTT by describing a novel approach for adopting one-time password (OTP) authentication schema, without the support of TLS. As also stated by the National Institute of Standards and Technology (NIST) guidelines (see the original document [6] and recent updates), OTP authentication allows the obtaining of strong authentication. In the case of not using the Lamport schema [7], i.e., when we want to avoid the problem of storing, protecting, and sharing a secret between prover and verifier, it is very important to have two channels, as independent as possible, for the transmission of the second-factor authentication (disposable) secrets.

With respect to the adoption of TLS, as suggested by the MQTT specification, our solution does not rely on security at lower layers, so reducing the impact in terms of energy consumption. Our solution also differs from the basic OTP authentication schema proposed for MQTT [8] since it adopts a blockchain as a second independent channel. Specifically, our proposal adopts Ethereum because it offers the powerful mechanism of smart contracts. In fact, an Ethereum smart contract ensures the correct execution of the protocol. Another important advantage of our solution is that despite the public availability of the data stored in a smart contract, the proposed solution preserves the privacy of users, mainly using hashes as user pseudonyms. The main advantages of our solutions are that it is capable of enhancing the security in MQTT by using a lightweight approach (without the adoption of TLS protocol at the transport layer), so restricting the work of the developers and the possibility to introduce security vulnerabilities.

The rest of the paper is structured as follows. Section 2 describes some related works and discusses the innovations of our solution with respect to the existing literature. Section 3 clarifies some preliminary concepts that are needed for the proposal. Section 4 describes the proposed solution. Section 5 reports the security analysis of the proposed solutions by discussing how it is robust with respect to possible malicious actions. Section 6 ends the paper by giving some conclusions and by suggesting possible future work.

## 2. Related Work

The ever-growing development of Internet of Things-based solutions leads to a set of security issues that must be taken into account. Current literature shows several works [9,10,11] addressing security challenges in IoT. Among them, in this paper, we focus our attention on the authentication problem [12,13]. In [14], the main authentication models for IoT are presented. However, our model is different as it uses blockchain as a secondary channel to transmit OTP codes that identify the attempts of access of devices.

Many works [15,16,17,18] adopt blockchain-based solutions to enforce the security of complex IoT applications. As an example, in [19], the authors present an interesting survey about the security problems in IoT and how blockchain can be used to face them. A blockchain-based solution to the authentication problem is discussed in [20]. However, this proposal contrasts only attacks where the attacker is in proximity to IoT devices, while our solution is more general and works with remote devices.

Another challenge is to provide an authentication mechanism which works with the most common message protocol for IoT, which is MQTT [21,22]. Security in MQTT has been deeply studied in the literature. For example, the study in [23] faces the confidentiality problem by using attribute-based encryption [24]. However, it has some drawbacks regarding inefficiency and complexity which should be avoided in IoT networks.

Regarding the authentication problem, we found the work proposed in [25] were similarly to our proposal. The authors present an authentication scheme using OTP for MQTT. With respect to our solution, their proposal requires the encryption of the exchanged messages. This approach has an extra effort that is similar to the adoption of the TLS protocol. In [26], we found another interesting proposal which implements an OTP-based authentication schema on MQTT. However, it is not specified how the device owner proves he/she is the legal user. Moreover, since the solution does not use blockchain, it is not possible to notarize the accesses of devices.

With respect to the proposals existing in the literature, our solution offers different novelties, which can be summarized in the following points: (1) blockchain is integrated with IoT application for authentication purposes without disclosing user identities, so preserving user privacy; (2) the proposed solution is totally compliant with the MQTT protocol since it preserves the standard messages of MQTT; and (3) the authentication is enforced by the OTP schema. The main advantage of adopting a blockchain resides in the possibility of having a public ledger trusted by its nature. In fact, with respect to the adoption of a public-key infrastructure, we do not need a trusted third party. Furthermore, a public-key infrastructure would require the use of a public-key cryptography that is not feasible for constrained devices (typical of the IoT domain). In particular, as will be clear in the rest of the paper, a central role in our proposal is assumed by smart contracts. One smart contract is published for each topic managed by each MQTT broker, so it is simple to verify if a user is authorized or not in that topic. We did not add this feature in the described proposal to keep authentication distinguished from authorization, to better integrate our approach to the standard MQTT protocol.

## 3. Background

This section reports some preliminary concepts that represent the background of our proposal. It gives some details about the MQTT protocol, also clarifying the roles of the different entities involved that are publishers, subscribers and brokers. Then, some preliminary concepts on blockchains and on Ethereum are also given.

### 3.1. The MQTT Protocol

Message Queuing Telemetry Transport (MQTT) is a lightweight messaging protocol designed for constrained devices. Usually, it runs at the application layer, on TCP (connection-oriented and reliable), but some variants as MQTT-S (for sensors networks) rely on UDP or other protocols. MQTT is often used as an alternative to HTTP in IoT networks. In fact, in those contexts where the bandwidth is limited and low-powered devices interact, HTTP might be too heavy to implement because of the high overhead. On the contrary, MQTT presents a very low overhead and small message size. Moreover, it provides an asynchronous communication model, thus it can be used even in case of devices with intermittent connectivity.

**Figure 1:**
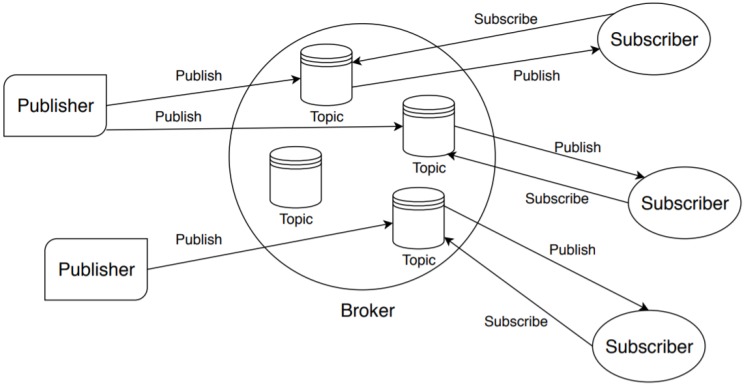
The main entities of the MQTT protocol.

MQTT is based on a publish–subscribe model and a client–server paradigm. The clients can be of two types: publishers (producers of the messages) and subscriber (consumers of the messages). They do not communicate directly between them, but through a server called broker. In detail, the broker runs different topics and receives the subscriptions on these topics from subscribers. When a publisher sends a message, it specifies the topic associated with the message and the broker forwards this message to the interested subscribers (i.e., those registered to the topic). The communication between clients (publishers or subscribers) and the broker takes place as part of a session. A session can be persistent or non-persistent. In a non-persistent session, if the connection is interrupted, all the subscriptions of the client to the various topics are not kept and the messages associated with such topics are lost. Moreover, when the client turns on and connects again with the broker, it has to re-subscribe to the topics, thus it cannot be efficient, especially when the clients are constrained devices with limited resources. Therefore, in these cases, a client can require a persistent session in which the broker stores (among other things) the subscriptions to topics and the messages for the client (even if the connection is interrupted and the client goes offline). When the client reconnects, all the undelivered messages associated with the topics it has subscribed to are forwarded by the broker. Non-persistent sessions are used when clients play, mainly, the role of publishers. The architecture of MQTT is depicted in Figure 1.

Initially, MQTT was developed to run on a private network, so security did not play a key role in the design of the protocol. However, today, MQTT is widely used in IoT networks, so new vulnerabilities and threats must be taken into consideration. In this paper, we focus our attention on the security aspects of MQTT; in particular, we investigate the authentication problem.

When a client wants to start a connection with the broker, it sends a CONNECT message. The basic authentication mechanism offered by MQTT provides two fields in the CONNECT message to transmit username and password to the broker. The broker evaluates the credentials and accepts or refutes the connection. The main problem is that these fields are sent in plain text and this exposes the client to an eavesdropping attack. However, it is possible to rely on secure transport protocol such as SSL or TLS to carry the message, but it has a price in terms of the light weight of the protocol.

A lighter approach to face the authentication problem is to use OTPs (passwords valid only for a session). Often OTPs are used as part of two-factor identification where the credentials of the client (username and password) are the first factor and OTPs are the second factor. However, OTPs cannot be sent on the same channel (MQTT messages) used for the credentials. Therefore, in this paper, we rely on blockchain as a separate channel to implement a two-factor authentication model for MQTT.

### 3.2. Blockchain

The objective of blockchain is to deploy a peer-to-peer (decentralized) network that keeps track of the occurrence of events. In a classical bank transaction, Alice sends *X* dollars to Bob and the bank guarantees the correctness of the transfer. In this way, users must, necessarily, trust the bank. There are two problems. First, recent events have shown that financial institution may be malicious. Moreover, we have no guarantees that the bank’s servers do not go down compromising the entire network. Blockchain resolves both the problems by implementing a peer-to-peer network that does not rely on a third trusted party to validate the transactions. Actually, blockchain can store any type of data, not just monetary transactions. However, currently, all implemented blockchain are used as a means to exchange value and they develop their own cryptocurrency. Cryptocurrencies are also used to reward users who contribute to the maintenance of the network.

Some of the main features that blockchain offers are:

- No third trusted party is necessary to maintain the network.
- The transactions must be validated and cannot be modified after they have been approved.
- Users cannot repudiate a transaction that they had generated.
- Users must prove they own the currency they spend.
- It shall not be possible to create currency from scratch. It is necessary to follow a legal protocol.
- Anyone can access and verify the transactions stored in the blockchain.
- The transactions must remain anonymous.

Regarding this latter point, for example, Bitcoin guarantees a pseudo-anonymity because, even if the identity of a user is not revealed, his/her transactions are linkable and it is possible to follow the flows of money.

The idea behind the blockchain is to have a single public ledger, shared by all nodes of the network that stores all the transactions in a way that they are easily checkable by anyone. All nodes maintain a copy of this ledger and can contribute to its construction by adding their own transactions or the transactions of other nodes. The first problem is: if each node has its own copy of the ledger, how can we get everyone to agree on what the right ledger is? We need a consensus mechanism that ensures that the nodes reach an agreement about the status of the network. In particular, a group of nodes exists called miners, which verify the transactions from different users and aggregate them into blocks. Then, the miners send, in broadcast, such blocks and each node can verify them. If the verification succeeds, the nodes add these blocks to its copy of the ledger. Therefore, in short, a blockchain is a chain of blocks of transactions. The word “chain” is used because each block also contains a pointer (i.e., the hash) to the previous block. This means that it is not possible to modify a block without modifying all subsequent blocks. Actually, to create a block is not just to aggregate transactions, but it also needs to win a cryptographic puzzle. If *n* is the current number of blocks in the chain, all miners compete to create the (n+1)th block and the first that wins the puzzle receives a reward. This challenge is called “proof of work” and it is necessary to demonstrate that miners have spent a huge computational effort in order to "mine" a block and thus, they are trustworthy. In fact, to win the game before the others, a malicious node should have more computational power than the rest of the network. The reward represents the incentive (along with the fees of the transactions) for the miners, to contribute to the construction of the blockchain. In this paper, we refer to the Ethereum blockchain which supports smart contracts. A smart contract is an entity stored in the blockchain, thus immutable, that is activated when it is triggered by a transaction of a user or by a message of another smart contract. The contract code is executed by the Ethereum virtual machine (EVM) and consists of a series of instructions written in a Turing complete programming language which allow the programmer to write arbitrary contracts for any situation. For example, the smart contracts are suitable in those cases where, in exchange for a sum of money, a customer requires digital service and the trustworthiness of the service provider or of the customer is not guaranteed. The smart contracts can ensure that the service provider receives the money and the consumer receives the service. A smart contract is executed when a user generates a transaction where the recipient’s address is the smart contract and miners try to include this transaction, in a block. Once the block is generated, the code of the smart contract is run, again, by all users, who verify the block and add it to the blockchain.

## 4. The Proposed Solution

The adoption of OTP authentication into MQTT represents a lightweight approach to face the authentication problem. This section describes in detail the proposed solution, which relies on the use of the Ethereum blockchain. Our solution reduces the energy consumption of IoT devices since they need to perform a few simple operations. Moreover, authentication is realized without the adoption of the TLS at the transport layer. The core of our solution resides inside Ethereum, and in particular in a smart contract that we developed. As will be clear in the rest of this paper, a solution that does not employ a smart contract suffers from serious drawbacks. We remark that our solution offers only authentication in MQTT, hence any other feature allowed in real-world adoptions of MQTT is also allowed with our authentication schema, including further authentication methods that can be combined with the proposed solution.

Furthermore, as in the general case of an MQTT-based architecture, we do not require the broker to reside in a specific physical location. For example, it may run on a specific host or reside in the cloud.

**Figure 2:**
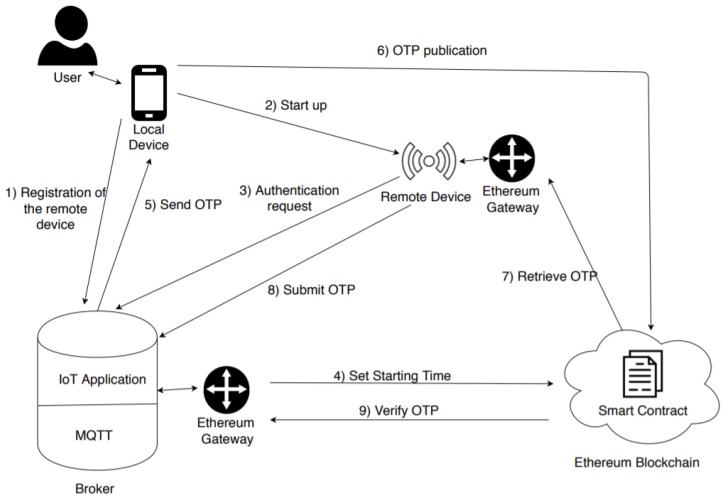
Realistic architecture of the proposed model.

Figure 2 depicts the entities of which the system is composed. We provide a brief overview of the authentication procedure. It will be discussed in more detail below. In a preliminary phase, a user registers a remote device, which she/he owns, with the broker. When such a device needs to authenticate (triggered by the user), it sends an authentication request to the broker. The broker calls a function of a smart contract (by generating a transaction) which starts a timer. Meanwhile, it generates an OTP and sends it to the user. This latter publishes the OTP on the blockchain and demonstrates that he/she has started the authentication request. When the OTP is published, the user calls another function on the smart contract which verifies that the OTP has not expired. If the OTP is valid, the remote device retrieves it by the blockchain and sends it to the broker. Finally, the broker retrieves the OTP (and other information) from the blockchain and verifies the correctness of the procedure. Please note that in a realistic scenario, the devices and the broker do not communicate, directly, with the Ethereum blockchain, but through an Ethereum gateway. Moreover, over the classic MQTT software, the broker has high-level software called IoT application, which is in charge of carrying out some operations of the authentication procedure: generation of the OTP, verification of the correctness of the latter, communication with the Ethereum gateway, and so on.

**Figure 3:**
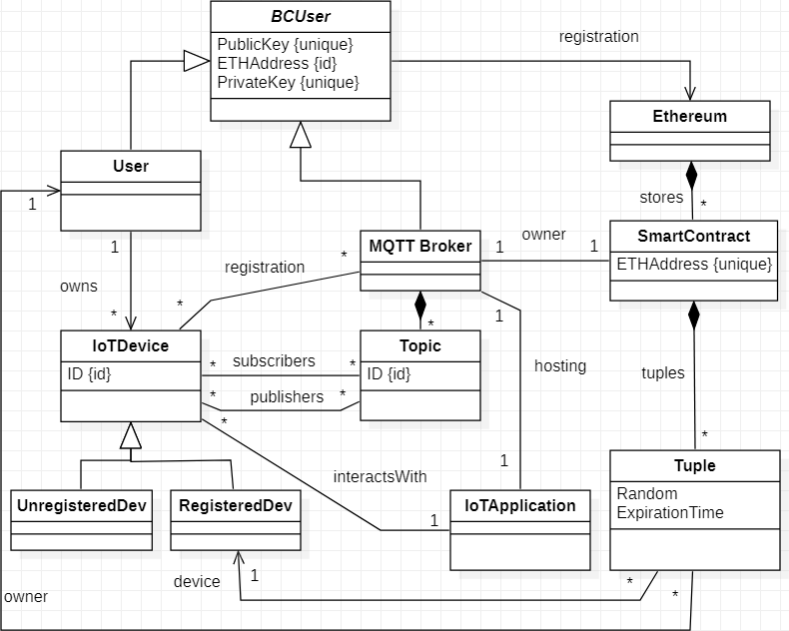
Domain model of our solution.

Starting from the described scenario and before the detailed description of the proposed authentication procedure, it is necessary to extract the domain model of our solution in order to formalize the concepts needed in the following. The domain model is depicted in the UML class diagram of Figure 3.

In this diagram, the concepts are depicted as UMLclasses and relationships are depicted as UML associations.

The main class of this model is IoTApplication, which represents a generic application in the IoT domain. Each IoT application is hosted by an MQTT broker and interacts with a set of IoT devices. Hence the corresponding class in the domain model is associated with the classes MQTTBroker and IoTDevice. The former models the concept of an MQTT Broker while the latter models whatever IoT device is involved in the functionalities offered by the IoT application. Each IoTDevice is identified by an ID (note that from now on, we consider the IPv6 address of the device as its identifier). The IoTDevice is connected to the class Topic with two different associations, which model the roles of publishers and subscribers they can play. As we will describe, the devices must be registered at the MQTT broker. Hence, the class IoTDevice is derived in two different classes in the domain model, i.e., UnregisteredDev and RegisteredDev. On the other hand, each device is owned by a User. The class User is generalized in the class BCUser, which represents a generic Ethereum user. It is characterized by an Ethereum address (identifier of the class) and a couple of keys (public and secret). In our approach, both a physical User and the MQTT Broker are possible users of the Ethereum blockchain; in fact, they inherit from the class BCUser. On the right of the model, we reported the concept of the Ethereum blockchain, represented by the homonym class, which stores a set of SmartContracts. Each Smart Contract has an Ethereum address and, for our solution, contains a set of tuples—one for each device.

After the complete formalization of the concepts inside our domain, we are now ready to describe the authentication process. As said before, our solution requires that every user is an Ethereum user as well as the MQTT broker. Furthermore, each device must be registered at the MQTT broker. Hence, the preliminary two steps of our solution are the following.

The first step requires that the owner *u* of a set of devices associates her/his identity with her/his Ethereum address $ETH_{u}$. To accomplish this task, *u* sends the tuple $\langle PK_{u},ETH_{u}^{SK_{u}}\rangle$ to the broker, where $PK_{u}$ is the *u*’s public key and $ETH_{u}^{SK_{u}}$ is the signature of $PK_{u}$ generated with the Ethereum private key $SK_{u}$. Consider that in Ethereum, the address is represented by the first 20 bytes of the hashed public key, hence the MQTT broker can open the $ETH_{u}^{SK_{u}}$ by retrieving $ETH_{u}$ and then it can check the correspondence between $ETH_{u}$ and $PK_{u}$. At the end of this step, the broker can link the identity of the user *u* with an Ethereum address $ETH_{u}$. Theoretically, the procedure of generation of the Ethereum addresses from the public keys could lead to the generation of two equal addresses. However, according the birthday attack, in a realistic complex application scenario where the number of users is around $10^{9}$, the probability of a collision is lower than $10^{-30}$. Hence, we can conclude that the probability of having colliding Ethereum addresses is negligible.

During the second step, the MQTT broker publishes a smart contract that stores a set of tuples whose single entry is of the form: 〈private_deviceID,owner_address,random,expiration_time〉, where private_deviceID is the hashed IP address of a device owned by a user, owner_address is the Ethereum address of that user, random is the random value used for OTP authentication, and expiration_time defines the expiration time of an OTP, after its generation. Therefore, for each registered user *u* who performed the Ethereum association, and for each device owned by *u* with IPv6 address *d*, a tuple $\langle ETH_{u},h\left(d\right),\_,\_\rangle$ exists in the smart contract, where h(d) denotes the application of a secure hash function on the IPv6 address *d*. Observe that since h(d) is not-reversible because IPv6 addresses are 128 bits, and the hash function *h* is assumed to be secure, the privacy of the user is not violated even though the storage of the smart contract is public. In particular, we refer to the hash function SHA-256, which is considered currently irreversible.

These tuples, in Ethereum smart contracts, are realized through a mapping, where the key is represented by the private_deviceID and the data is represented by a struct made of the remaining three fields.

After these preliminary steps, the system is ready to allow for secure device OTP authentications. We distinguish between the authentication of local devices and of remote devices. This includes, indirectly, the authentication of publishers and subscribers in MQTT. In fact, usually, an IoT publisher is a sensing device which must be remotely authenticated by a user. On the other hand, a subscriber is an actuator device, locally used by the user to read the data published on specific topics.

**Figure 4:**
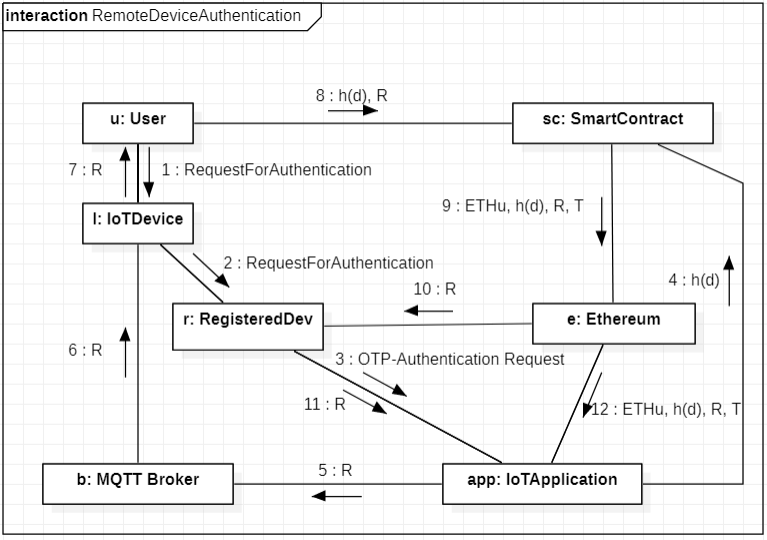
Authentication of a remote device.

We start from the description of the authentication procedure of a remote device. In this case, the user, in order to authenticate the owned remote IoT device, needs an auxiliary local IoT device (not necessarily registered to the MQTT broker) which is able to communicate with the remote device and with the broker. The UML communication diagram in Figure 4 describes the sequence of messages needed to authenticate the remote IoT device acting as publisher. The process starts by the user *u* which contacts, through her/his local device (namely *l*), the remote device (namely *r*) with IPv6 address *d* (Messages 1–2) asking it to perform the procedure of authentication. When *r* receives the request of *u*, it contacts the IoT Application running on the broker (Message 3). This latter extracts a random *R* from a strong True Random Number Generator (TRNG) and calls a function of the smart contract by sending a transaction from its Ethereum address $ETH_{BR}$ towards the smart contract, which has the Ethereum address $ETH_{SC}$ (Message 4), which contains h(d) as function parameter. By executing this function, the entry $\langle ETH_{u},h\left(d\right),\_,\_\rangle$ changes to $\langle ETH_{u},h\left(d\right),\_,T_{gen}\rangle$ where $T_{gen}$ represents the OTP generation time. From the other side, the value *R* representing the OTP is sent to the broker and shown to the user by *l* (Messages 5–6–7). The user reads *R* on her/his device and calls a different function of the smart contract by sending a transaction from her/his Ethereum address $ETH_{u}$ (Message 8), which contains the couple (R,h(d)), as function parameters. This function retrieves the entry $\langle ETH_{u},h\left(d\right),\_,T_{gen}\rangle$ from the smart contract storage, verifies the temporal validity of the OTP by evaluating the difference between the current time and $T_{gen}$, and updates the entry by adding the value *R* if the OTP is not expired. Hence, the final form of the entry is $\langle ETH_{u},h\left(d\right),R,T\rangle$, where *T* represents the current time. Please note that the delay introduced by the mining of the transactions on the block chain does not impact the validity of our solution. In fact, after Message 8 the smart contract stores the timestamp of the method invocation that is not affected by the following mining time. Hence, the temporal validity considered by the smart contract is related to the net interval of time between Messages 4 and 8 and it is not affected by mining delays.

After this last operation, the smart contract publishes an event on the Ethereum blockchain containing the entire entry (Message 9). Finally. the remote device *r* recovers the OTP *R* from the list of Ethereum events, and sends it to the IoT application (Messages 10–11). This latter verifies the correctness of *R* and accesses the Ethereum event list retrying the public entire entry (Message 12). In particular, the IoT application verifies that *R* is still valid; h(d) and $ETH_{u}$ represents the proper user under authentication and checks the freshness of the timestamp *T*. This last step is needed to check the correct execution of the entire authentication procedure. In particular, we recall here that in our hypothesis the remote device *r* is under the control of the user *u*. Hence, if no access to the blockchain is performed, the IoT application has no evidence that the user has correctly executed the entire procedure.

**Figure 5:**
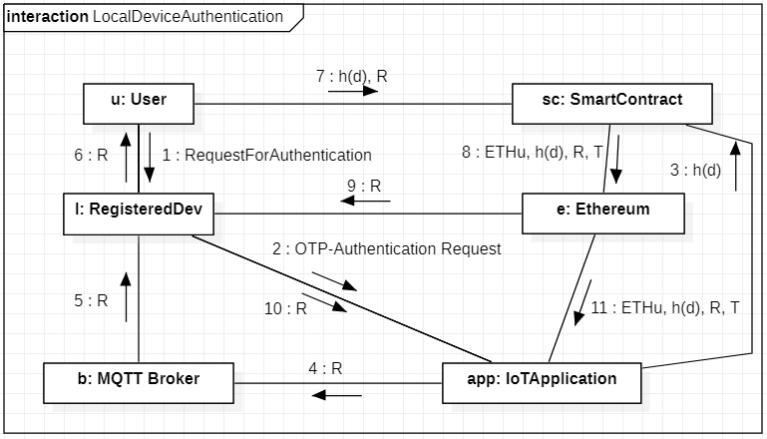
Authentication of a local device.

The authentication of a local device is simpler than the previous since the process includes a single device. Figure 5 depicts the UML communication describing this process. Initially, the user *u* contacts his/her local device *l* (with IPv6 address *d*) to start the authentication procedure (Message 1). In turn, *l* contacts the IoT application (Message 2). This latter extracts a random *R* from a TRNG and calls a function of the smart contract by sending a transaction from its Ethereum address $ETH_{BR}$ towards the smart contract, which has the Ethereum address $ETH_{SC}$ (Message 3), which contains h(d) as function parameter. By executing this function, the entry $\langle ETH_{u},h\left(d\right),\_,\_\rangle$ changes to $\langle ETH_{u},h\left(d\right),\_,T_{gen}\rangle$ where $T_{gen}$ represents the OTP generation time. From the other side, the value *R* representing the OTP is sent to the broker and shown to the user by *l* (Messages 4–5–6). The user reads *R* on her/his device and calls a different function of the smart contract by sending a transaction from her/his Ethereum address $ETH_{u}$ (Message 7), which contains the couple (R,h(d)), as function parameters. This function retrieves the entry $\langle ETH_{u},h\left(d\right),\_,T_{gen}\rangle$ from the smart contract storage, verifies the temporal validity of the OTP by evaluating the difference between the current time and $T_{gen}$, and updates the entry by adding the value *R* if the OTP is not expired. Hence, the final form of the entry is $\langle ETH_{u},h\left(d\right),R,T\rangle$, where *T* represents the current time. Consequently, the smart contract publishes an event on the Ethereum blockchain containing the entire entry (Message 8). Finally. the local device *l* recovers the OTP *R* from the list of Ethereum events, and sends it to the IoT application (Messages 9–10). The latter verifies the correctness of *R* and accesses to the Ethereum event list retrying the public entire entry (Message 11). In particular, the IoT application verifies that *R* is still valid, h(d) and $ETH_{u}$ represents the proper user under authentication and checks the freshness of the timestamp *T*.

Before concluding this section, it is important to say that the deployment and execution costs of the smart contract are entirely accounted to its owner that in our solution, is represented by the broker. These costs are rather low, and have been evaluated around 20Gwei.

## 5. Security Analysis

The objective of this section is to analyze the security properties offered by our solution. We first define our threat model.

**Assumptions.**

Our threat model relies on several realistic assumptions.

**A1**  : The OTP generation is based on a secure random generation. Therefore, the generated number is not guessable by any party.
**A2**  : The MQTT broker is not malicious, but it is not immune from attacks on integrity of its databases.
**A3**  : The smart contract is bug-free, and any out-of-chain interaction performs correctly, and its integrity is guaranteed.
**A4**  : The secrecy of private keys corresponding to Ethereum addresses cannot be violated.
**A5**  : Ethereum transactions/addresses are managed in such a way (e.g., mixnet services, disposable addresses, etc.) that even by considering possible available background information, de-anonymization is infeasible.
**A6**  : The adopted cryptographic hash function is secure, in the sense it is robust against pre-image, second pre-image and collision attacks. As a matter of fact, to the best of the current knowledge, SHA-256 is an example of hash function fulfilling this assumption.

**Adversaries.**

The adversary in our threat model is any external party.

**Security Properties.**

Our protocol is required to satisfy the following three properties:

**SP1**  : *(Not Impersonation)* No adversary can impersonate the legitimate user in the authentication process.
**SP2**  : *(Privacy)* No privacy leakage regarding device owners occurs.
**SP3**  : *(Accountability)* The responsibility of any action can be attributed to the actual actor.

We now analyze the attacks compliant with the above threat model and show that security properties are still satisfied.

**AA1**  : The attacker intrudes into the database of the MQTT broker and changes the association of a generated OTP in favor of an impostor.
**AA2**  : A legitimate user or a legitimate device keeps the random *R* and tries to re-use it (replay attack).
**AA3**  : The attacker tries to disclose the identity of the user who has performed the authentication procedure through a device or tries to disclose the IPv6 address of such device.
**AA4**  : The attacker declares an access never actually occurred or repudiates a performed access.

The assumptions **A1** and **A2** are essentials for the entire protocol, as previously explained in the description of the model. Considering the attack **AA1**. When a legitimate user *u* performs an authentication request, the broker stores the random *R* and the IPv6 address *d* of the device which originates the request until it verifies these values match with the values recovered by the blockchain. If the attacker alters *R* or *d* in the database of the broker, it has to change the corresponding value of the entry $\langle ETH_{u},h\left(d\right),R,T\rangle$. Due to assumptions **A3**, the data stored on the smart contract cannot be altered. Thus, then only option for the attacker is to generate a legal Ethereum transaction originated from the address $ETH_{u}$, where *R* or h(d) is different by the legal value (i.e., the attacker impersonates the legitimate user). This is impossible due the assumption **A4**, since the private key of the legitimate user is kept secret. This shows that the property **SP1** is verified. The attack **AA2** is easily contrasted through the expiration time associated with the random *R*. In fact, in case a user tries to use *R* after its validity time, the smart contract detects it. On the other hand, if a device does not use *R* within an appropriate time frame, the IoT application does not allow authentication. Regarding **AA3**, the only public information linked to the users are their Ethereum addresses. The association identity Ethereum address is kept only by the broker. Thus, if this latter is not compromised, since the identities of the users cannot be retrieved by any de-anonymization (Assumption **A5**), the privacy of the users is preserved (security property **SP2**). Furthermore, regarding the IPv6 addresses, only their digests are published on the blockchain. Since their domain is large enough (128 bits) and the hash function is secure (Assumption **A6**), they cannot be disclosed. Finally, for the attack **AA4**, all the accesses to the devices are stored on the blockchain in form of pseudonyms. In the case of misbehavior, the information stored by the broker is enough to reveal and account each authentication attempt to a user and to her/his specific device. This shows that the property **SP3** is guaranteed.

## 6. Conclusions

This paper proposes an extension of the MQTT messaging protocol, adding OTP authentication by using Ethereum as a second communication channel. With respect to the native authentication mechanism provided by the MQTT protocol, this solution ensures secure authentication, preserving privacy and allowing for accountability. Consider that the standard MQTT protocol is based on the simple transmission from the client to the MQTT broker of a message containing a username and password as plain text.

With respect to the adoption of TLS at the transport layer, our solution could deal with low-power devices typical of the IoT domain. Our solution introduces into the protocol the most lightweight mechanism to implement strong authentication, i.e., OTP. Observe that in our solution the MQTT broker is in charge of generating a secure challenge (i.e., to execute either a PRNG or a TRNG algorithm), thus unloading the work of devices. Even if a naive inclusion of the OPT mechanism into the MQTT messaging would keep open some vulnerabilities, our solution overcomes the well-known drawbacks thanks to the adoption of Ethereum and smart contracts. Moreover, increased levels of privacy and accountability are obtained by relying on the trust offered by the blockchain.

As a final consideration, we can observe that the usage of blockchain and smart contracts provides benefits in terms of accountability and forensics, because all the authentication events are stored immutably on chain. If needed in the specific application context, further transactions could be included (for instance, a confirmation transaction generated at the end of the authentication by the MQTT broker), to enforce the accountability forensic information available in the blockchain.

In terms of future work, we plan to perform an experimental validation of the proposal, by also verifying the costs of the smart contract operations.

## Abbreviations

The following abbreviations are used in this manuscript:

HTTP	HyperText Transfer Protocol
IEC	International Electrotechnical Commission
ISO	International Organization for Standardization
MQTT	Message Queuing Telemetry Transport
NIST	National Institute of Standards and Technology
OTP	One-time password
TCP	Transmission Control Protocol
TLS	Transport Layer Security
TRNG	True random number generator
UDP	User Datagram Protocol
UML	Unified Modeling Language
UTF	Unicode Transformation Format

## References

[1] ISO/IEC 30141:2018 (2018). Internet of Things (loT) — Reference Architecture. https://www.iso.org/standard/65695.html.

[2] Hammi M.T., Hammi B., Bellot P., Serhrouchni A. (2018). Bubbles of Trust: A decentralized blockchain-based authentication system for IoT. Comput. Secur..

[3] Deering S., Hinden R. (2017). Internet Protocol, Version 6 (IPv6) Specification. IETF Std. RFC8200. https://tools.ietf.org/html/rfc8200.

[4] Banks A., Gupta R. (2014). MQTT Version 3.1.1. OASIS Standard. http://docs.oasis-open.org/mqtt/mqtt/v3.1.1/os/mqtt-v3.1.1-os.html.

[5] Banks A., Briggs E., Borgendale K., Gupta R. (2019). MQTT Version 5.0. OASIS Standard. https://docs.oasis-open.org/mqtt/mqtt/v5.0/os/mqtt-v5.0-os.html.

[6] Burr W.E. (2006). NIST Special Publication 800-63: Electronic Authentication Guideline. http://csrc.nist.gov/publications/nistpubs/800-63-1/SP-800-63-1.pdf.

[7] Lamport L. (1981). Password authentication with insecure communication. Commun. ACM.

[8] Tripathy B., Anuradha J. (2017). Internet of Things (IoT): Technologies, Applications, Challenges and Solutions.

[9] Zhang Z.K., Cho M.C.Y., Wang C.W., Hsu C.W., Chen C.K., Shieh S. IoT security: Ongoing challenges and research opportunities. Proceedings of the 2014 IEEE 7th International Conference on Service-Oriented Computing and Applications.

[10] Weber R.H. (2010). Internet of Things–New security and privacy challenges. Comput. Law Secur. Rev..

[11] Mahmoud R., Yousuf T., Aloul F., Zualkernan I. Internet of things (IoT) security: Current status, challenges and prospective measures. Proceedings of the 2015 10th International Conference for Internet Technology and Secured Transactions (ICITST).

[12] El-Hajj M., Chamoun M., Fadlallah A., Serhrouchni A. Analysis of authentication techniques in Internet of Things (IoT). Proceedings of the 2017 1st Cyber Security in Networking Conference (CSNet).

[13] Ranjan A., Somani G., Mahmood Z. (2016). Access Control and Authentication in the Internet of Things Environment. Connectivity Frameworks for Smart Devices.

[14] Zhang Z.K., Cho M.C.Y., Shieh S. Emerging security threats and countermeasures in IoT. Proceedings of the 10th ACM Symposium on Information, Computer and Communications Security.

[15] Christidis K., Devetsikiotis M. (2016). Blockchains and smart contracts for the internet of things. IEEE Access.

[16] Dorri A., Kanhere S.S., Jurdak R. (2016). Blockchain in internet of things: Challenges and solutions. arXiv.

[17] Kshetri N. (2017). Can blockchain strengthen the internet of things?. IT Prof..

[18] Ouaddah A., Abou Elkalam A., Ait Ouahman A. (2016). FairAccess: A new Blockchain-based access control framework for the Internet of Things. Secur. Commun. Networks.

[19] Khan M.A., Salah K. (2018). IoT security: Review, blockchain solutions, and open challenges. Future Gener. Comput. Syst..

[20] Wu L., Du X., Wang W., Lin B. An out-of-band authentication scheme for internet of things using blockchain technology. Proceedings of the 2018 International Conference on Computing, Networking and Communications (ICNC).

[21] Niruntasukrat A., Issariyapat C., Pongpaibool P., Meesublak K., Aiumsupucgul P., Panya A. Authorization mechanism for MQTT-based Internet of Things. Proceedings of the 2016 IEEE International Conference on Communications Workshops (ICC).

[22] Calabretta M., Pecori R., Veltri L. A Token-based Protocol for Securing MQTT Communications. Proceedings of the 2018 26th International Conference on Software, Telecommunications and Computer Networks (SoftCOM).

[23] Singh M., Rajan M.A., Shivraj V.L., Balamuralidhar P. Secure MQTT for Internet of Things (IoT). Proceedings of the 2015 Fifth International Conference on Communication Systems and Network Technologies.

[24] Goyal V., Pandey O., Sahai A., Waters B. Attribute-based encryption for fine-grained access control of encrypted data. Proceedings of the 13th ACM conference on Computer and communications security.

[25] Jayan A., Balasubramani A., Kaikottil A., Harini N. (2019). An enhanced scheme for authentication using OTP and QR code for MQTT protocol. Int. J. Recent Technol. Eng..

[26] Gantait A., Patra J., Mukherjee A. (2018). Securing IoT Devices and Gateways. https://developer.ibm.com/articles/iot-trs-secure-iot-solutions1/.

